# Occurrence of halonaphthoquinone disinfection byproducts in Shanghai’s drinking water and the hepatotoxic effects on lipid accumulation

**DOI:** 10.3389/fpubh.2026.1831866

**Published:** 2026-07-07

**Authors:** Zhenzhen Tian, Yongqing Diao, Jielan Hu, Yuxin Li, Xi Yu, Xia Wang

**Affiliations:** 1 Key Laboratory of the Public Health Safety, Ministry of Education, Department of Environmental Health, School of Public Health, Fudan University, Shanghai, China; 2 Shanghai Qingpu District Center for Disease Control and Prevention, Shanghai, China

**Keywords:** disinfection by-products, halonaphthoquinones, high performance liquid chromatography–tandem mass spectrometry, lipid accumulation, oxidative stress

## Abstract

**Introduction:**

In recent years, aromatic disinfection by-products (DBPs) have attracted widespread concern due to their elevated concentration and significant toxicity. Recent studies have identified a new class of DBPs, halonaphthoquinones (HNQs), during drinking water disinfection processes, which exhibit both high occurrence levels and considerable toxicological risks.

**Methods:**

This study established a liquid–liquid extraction coupled with HPLC-MS/MS method to determine HNQs levels in drinking water. In July 2024, source and finished water samples were collected from three drinking water treatment plants (Plants A, B, and C) in Shanghai, which source their water from the Q and J reservoirs. A total of 9 source and 9 finished water samples were collected in each plant. Based on the HNQs concentrations detected in finished water, the effects of mixed HNQs exposure on lipid accumulation in HepG2 human hepatoma cell line in vitro were investigated.

**Results:**

In finished water, MCNQ showed the highest levels, averaging 8.7–13.05 ng/L and peaking at Plant A (13.05 ng/L, *p* < 0.05). Followed by MBNQ, with a mean range of 1.37–13.89 ng/L and a peak at Plant B (13.89 ng/L, *p* < 0.05). Combined exposure to HNQs in drinking water can induce lipid accumulation in HepG2 cells. Under HF culture conditions, HepG2 cells exhibited lipid accumulation even at shorter exposure time and lower concentrations of HNQs, suggesting that HF and HNQs may act synergistically to promote hepatocytic lipid accumulation. Mixed exposure to HNQs can induce oxidative stress in HepG2 cells, possibly by inhibition of *IPMK/AMPK/SIRT1/PGC-1α* pathway genes expression, which may reduce mitophagic clearance capacity, thereby disrupting the cellular redox balance and promoting lipid accumulation.

**Discussion:**

These results suggest that HNQs may be important risk factors for metabolic dysfunction-associated fatty liver disease (MAFLD), and further observation and validation of the impact of HNQs on MAFLD are needed in the future.

## 1 Introduction

Drinking water contamination is a growing global concern. While disinfection is essential for eliminating pathogens, it can lead to the formation of disinfection by-products (DBPs) through reactions between disinfectants and natural organic matter (NOM) (1, 2). The intensified use of disinfectants during the COVID-19 pandemic (3) may have further elevated DBP levels in aquatic environments, potentially impacting human health (4). Over 700 DBPs have been identified, yet only about 100 have been thoroughly investigated for their formation, occurrence, and toxicity (5).

Research on drinking water DBPs has long focused on aliphatic species, such as trihalomethanes (THMs) and haloacetic acids (HAAs). In recent years, however, aromatic DBPs have drawn growing concern. Compared with aliphatic DBPs, aromatic DBPs exhibit greater chemical stability and higher toxicity, posing a more significant potential threat to both aquatic ecosystems and human health (6). Recent studies have detected halonaphthoquinones (HNQs), an emerging class of DBPs, in drinking water. Concentrations up to 136.7 ng/L were reported in seven treatment plants in Zhejiang Province, China (7). Although data on HNQs occurrence remain limited, polycyclic aromatic hydrocarbons (PAHs) have been considered as critical precursors for HNQs formation during water disinfection (7). PAHs are a class of ubiquitous environmental organic pollutants consisting of two or more fused aromatic rings, which can persist in the atmosphere (8), soil (9, 10), and various freshwater (11, 12) and marine environments (13, 14). This widespread PAH contamination in source waters thus provides a substantial material basis for the formation of aromatic DBPs such as HNQs. However, research on HNQs occurrence in drinking water remains scarce, and no targeted regulatory measures have yet been established.

As the primary site for xenobiotic metabolism, the liver is highly susceptible to environmental pollutants and serves as the body’s frontline defense against harmful chemicals. Disruption of hepatic lipid metabolism is a common pathological response to exogenous compounds, potentially leading to metabolic dysfunction-associated fatty liver disease (MAFLD) (15, 16). Among aromatic disinfection by-products, halobenzoquinones (HBQs) exhibit pronounced cytotoxicity attributed to their strong redox activity and capacity for nucleophilic addition reactions (17–20). Mechanistic studies have demonstrated that HBQs elevate reactive oxygen species (ROS), disrupt glutathione (GSH) homeostasis, and inhibit antioxidant enzymes such as superoxide dismutase (SOD) and catalase (21, 22).

Halonaphthoquinones (HNQs), which share structural similarities with HBQs, may induce comparable toxic effects. This concern is supported by recent findings that HNQs exhibit IC50 values ranging from 3.17 to 13.18 μM, comparable to that of iodoacetic acid (2.95 μM), one of the most potent aliphatic DBPs (7). The *AMPK/SIRT1/PGC-1α* signaling pathway, a key regulator of energy metabolism, has been shown to restore mitochondrial function and provide protection against oxidative stress, inflammation, and apoptosis when activated (23, 24), suggesting a potential mechanistic link to HNQ-induced hepatotoxicity.

Due to the limited availability of analytical methods and reference standards for HNQs—particularly iodinated congeners—this study developed and applied a liquid–liquid extraction (LLE) coupled with HPLC-MS/MS method to quantify four HNQs in drinking water: 2-chloro-1,4-naphthoquinone (MCNQ), 2-bromo-1,4-naphthoquinone (MBNQ), 2,3-dichloro-1,4-naphthoquinone (DCNQ), and 2,3-dibromo-1,4-naphthoquinone (DBNQ). Source water and finished water samples were collected from three drinking water treatment plants supplied by Shanghai’s Q and J Reservoirs to characterize HNQ contamination. Based on the measured concentrations in finished water, a HepG2 cell model was established to investigate, for the first time, the combined effects of HNQs mixture exposure on hepatocyte lipid accumulation and its mechanistic basis. This work provides essential data on the occurrence and health implications of HNQs in drinking water, informing future pollution control and regulatory efforts.

## 2 Materials and methods

### 2.1 Reagents

All reagent-grade chemicals and biological reagents used in this study were obtained from commercial suppliers (see Supplementary Text S1).

### 2.2 Sample preparation

In this study, liquid–liquid extraction was employed to pre-concentrate HNQs from water samples prior to HPLC-MS/MS analysis. A 15 mL volume of n-hexane was used as the extraction solvent, and the pH of the water sample was adjusted to 5 before extraction. Detailed information regarding the pretreatment procedure for the analysis of HNQs in water samples is provided in Text S2.

### 2.3 Sample collection

During the wet season in July 2024, we collected source water and finished water from three drinking water treatment plants (DWTPs) in Shanghai, China. The samples were collected in 500 mL amber glass bottles with no headspace. At each DWTP, sampling was performed in triplicate daily over three consecutive days. Although the treatment processes of the three DWTPs differ slightly (see Supplementary Figure S2), all samples were transported to the laboratory on ice packs and stored at 4 °C.

### 2.4 HPLC-MS/MS analysis

The separation and quantification of HNQs were performed using a Shimadzu high-performance liquid chromatography (HPLC) system (LC-20A) coupled with an AB SCIEX QTRAP 5500 triple quadrupole ion-trap tandem mass spectrometer, referring to the detection method described by Jiang et al. (7). Separation was achieved using a Waters ACQUITY UPLC BEH C18 column (2.1 mm × 100 mm, 1.7 μm). The target analytes were quantified using multiple reaction monitoring (MRM) in negative electrospray ionization mode. Identification and quantification were performed according to the retention times and peak areas of the corresponding ion pairs. Detailed chromatographic and mass spectrometric conditions were provided in Supplementary Text S4.

### 2.5 Quality control

To monitor potential contamination during transport and handling, travel blanks prepared with ultrapure water were analyzed concurrently with the field samples. System performance and cross-contamination were assessed by injecting analysis blanks, composed of 70% methanol and 30% water, into the HPLC-MS/MS system after every three sample injections. Prior to each sample analysis, an external standard calibration curve for the mixed HNQs was established. Quality assurance and quality control measures, including recoveries, relative standard deviations (RSD), method detection limits (MDL), method quantitation limits (MQL), and linear ranges, are detailed in Supplementary Text S5.

### 2.6 Cell culture and treatment

In this study, the cytotoxicity was assessed *in vitro* using the HepG2 cell line (human hepatoblastoma). HepG2 cells were purchased from the cell bank of the Typical Culture Preservation Committee of the Chinese Academy of Sciences. The HepG2 cells were cultured in DMEM supplemented with 10% (v/v) FBS and 1% (v/v) penicillin/streptomycin at 37 °C and 5% (v/v) CO_2_ atmosphere. Cells were maintained in Dulbecco’s Modified Eagle Medium (DMEM) containing 10% (v/v) fetal bovine serum (FBS) and 1% (v/v) penicillin/streptomycin, and cultured at 37 °C in a humidified atmosphere containing 5% (v/v) CO_2_.

To model high-fat (HF) conditions *in vitro*, cells were incubated with 0.1 mM sodium oleate (OA). The cells were treated with the mixed HNQs for 24, 48, and 72 h, either with or without the HF induction. For the lipid deposition assay, the following experimental groups were included: (1) negative control (0.5% DMSO), (2) HF group (0.1 mM OA), and (3) HNQs treatment groups. We prepared a mixture of the four HNQs at the maximum average concentration of the finished water from the three DWTPs, using multiples of the measured concentration—1 to 1,000-fold. The specific exposure doses are detailed in Supplementary Table S6.

### 2.7 Cytotoxicity assay and quantification of triglyceride analysis

HepG2 cells in the logarithmic growth phase were seeded into 96-well plates at 2 × 10^4^ cells per well and allowed to adhere for 24 h. All experiments were conducted with three independent biological replicates (*n* = 3), with each replicate having at least three technical replications. After 24 h, the medium was replaced with fresh medium containing various concentrations of HNQs, and the cells were cultured for an additional 24, 48, or 72 h, either with or without HF conditions. Cell viability was assessed by measuring total DNA content using NucBlue, and results were expressed as a percentage of the control cells. Intracellular triglyceride levels were quantified using the commercial reagent AdipoRed (25). The results were normalized to total DNA content, as quantified by the NucBlue assay (26). Cell viability and relative triglyceride content (RTC) were calculated using the following equation:

$$\text{Cell viability}=\frac{{\mathrm{FI}}_{\text{exptl}}-{\mathrm{FI}}_{\text{backgr}}}{{\mathrm{FI}}_{\text{blank}}-{\mathrm{FI}}_{\text{backgr}}}$$

$$\mathrm{RTC}=\frac{\begin{array}{l} \;({\mathrm{FI}}_{\mathrm{TG},\text{exptl}}-{\mathrm{FI}}_{\mathrm{TG},\text{backgr}})/\\ ({\mathrm{FI}}_{\mathrm{DNA},\text{exptl}}-{\mathrm{FI}}_{\mathrm{DNA},\text{backgr})} \end{array}}{\begin{array}{l} \;({\mathrm{FI}}_{\mathrm{TG},\text{blank}}-{\mathrm{FI}}_{\mathrm{TG},\text{backgr}})/\\ \left({\mathrm{FI}}_{\mathrm{DNA},\text{blank}}-{\mathrm{FI}}_{\mathrm{DNA},\text{backgr}}\right) \end{array}}$$

Where FI_exptl_, FI_backgr_, and FI_blank_ are the fluorescence intensities of the experimental group, background group, and blank control group, respectively. The measurements were repeated 3 times.

Oil Red O staining was performed to evaluate lipid droplet accumulation in HepG2 cells following exposure to HNQs under HF conditions. Briefly, cells were fixed with 4% paraformaldehyde for 10 min, washed, and then incubated with a 0.5% Oil Red O working solution for 30 min at room temperature. Subsequently, the cells were counterstained with hematoxylin for 2 min to visualize nuclei, rinsed thoroughly with distilled water, and observed under a light microscope for lipid droplet analysis.

### 2.8 Cellular ROS, SOD activity and GPx activity

HepG2 cells in the logarithmic growth phase were seeded into 6-well plates at 1 × 10^5^ cells per well and allowed to adhere for 24 h with 4 replicates. To evaluate HNQs-induced oxidative stress under HF conditions, HepG2 cells were incubated with 50, 100, 500, and 1,000-fold real-world concentrations of HNQs for 72 h. Cellular ROS levels were measured using a fluorometric assay kit, with fluorescence intensity recorded at excitation and emission wavelengths of 488 nm and 525 nm, respectively, on a microplate reader. The positive control group was Rosup from the kit diluted at a ratio of 1:1000 using serum-free medium. The SOD and enzymatic activities of glutathione peroxidase (GPx) were assayed using commercial kits strictly following the manufacturer’s instructions. Total protein concentrations in cell lysates were determined using the BCA Protein Concentration Assay Kit to normalize enzyme activities.

### 2.9 RNA extraction and quantitative reverse transcription PCR (qRT-PCR)

HepG2 cells were exposed to 50–1,000 times the realistic level of mixed HNQs found in Shanghai’s finished water for 72 h under HF conditions. Total RNA was extracted using the RNAeasy Isolation Kit with a spin column following the manufacturer’s instructions. After dilution in RNase-free H2O, RNA concentration was measured at 260 nm using Nanodrop (Thermo, Wilmington, DE), as shown in TextS6.

To investigate gene expression, HepG2 cells were exposed to 50–1,000 times the realistic concentrations of mixed HNQs detected in finished water from DWTPs for 72 h under HF conditions. Following exposure, total RNA was isolated using the RNAeasy Isolation Kit with spin column technology, strictly adhering to the manufacturer’s protocol. The purified RNA was eluted in RNase-free water, and its concentration was quantified by measuring absorbance at 260 nm using a NanoDrop spectrophotometer (Thermo, Wilmington, DE). The sequences of the primer pairs were listed in Supplementary Text S6.

### 2.10 Statistical analyses

Data processing and statistical analysis were conducted using GraphPad Prism software (version 10.4.1). To determine statistically significant differences between multiple groups, one-way analysis of variance (ANOVA) was performed. Following ANOVA, if a significant *F*-value (*p* < 0.05) was obtained, Dunnett’s multiple comparison test was applied to assess differences between each treatment group and the control group. All statistical tests were two-tailed, and the significance level was set at *α* = 0.05. Quantitative data were expressed as the mean ± standard deviation (SD).

## 3 Results and discussion

### 3.1 Establishment and optimization of HNQs detection methods in drinking water

Studies on the occurrence of HNQs in drinking water is currently limited. In previous studies, n-hexane has proven to be an effective extraction solvent for a range of organic pollutants, including polycyclic aromatic hydrocarbons (PAHs), with reported relative recoveries of 85.5–101.5% (27). Additionally, n-hexane has been successfully utilized for the extraction of polychlorinated biphenyls (28), phthalate esters (29), and bisphenols from aqueous matrices, yielding recoveries ranging from 84.3 to 119.8%. Considering the similar chemical structures and properties of HNQs to these well-studied contaminants, n-hexane was selected as the extraction solvent in the present work. To optimize the pretreatment procedure, the extraction performance of different volumes of n-hexane was systematically compared for the four target HNQs.

The efficiency of LLE was first optimized by varying the volume of n-hexane. Recovery rates increased with solvent volume, with 15 mL and 20 mL yielding the highest relative recoveries (>85%) for the four HNQs (Supplementary Figure S2a). To balance extraction performance with practical considerations, 15 mL of n-hexane was chosen as the optimal volume, as it minimized solvent use and reduced the time required for nitrogen evaporation. Subsequently, the effect of sample pH on extraction efficiency was investigated using the selected solvent volume. As illustrated in Supplementary Figure S2b, the highest and most consistent recoveries across all four HNQs were achieved at pH 5, which was therefore adopted for all subsequent analyses.

The results of validation of the analytical method were summarized in Supplementary Table S3. The spiked recoveries for the four HNQs ranged from 95.0 to 103.0%. The RSD for both inter-day and intra-day precision were consistently below 20% (Supplementary Table S4), and the method detection limits (MDL) were determined to be 0.02 ng/L-0.2 ng/L (Supplementary Table S2). When compared with solid-phase extraction (SPE), the LLE pretreatment technique employed in this study exhibited superior performance, including higher recovery rates and shorter processing times. Moreover, LLE yielded lower method detection and quantification limits.

### 3.2 Occurrence of HNQs in drinking water in Shanghai

The occurrence of HNQs in source water from the three DWTPs was summarized in Tables 1, 2. Overall, the concentrations of ∑HNQs were low across all source water samples. Among the four target analytes, only DCNQ was detected above the MDL, with a detection frequency of 100%. The mean concentrations of DCNQ in source water ranged from 0.18 ng/L to 0.50 ng/L. A comparison among the three DWTPs showed that the highest ∑HNQs level was recorded in DWTP C (0.5 ng/L), while the lowest was found in DWTP A (0.18 ng/L). The source water for the three DWTPs originates from different rivers: DWTP A from the Yangtze River, and DWTP B and DWTP C from the Huangpu River. The source water for the three DWTPs is supplied by the Q Reservoir and the J Reservoir. The Q Reservoir is sourced from the lower reaches of the Yangtze River, while the J Reservoir draws from the Huangpu River. Previous investigations have documented organic contamination in the Yangtze River and Huangpu River, with pollutant levels in the Huangpu River approximately twice those measured in the Yangtze estuary, which may be associated with the Huangpu River’s geographical location and surrounding anthropogenic activities.

**Table 1:** Detection rates of HNQs in source water and finished water of DWTP A, B and C.

Compound	Source water (%)				Finished water (%)			
	A	B	C	Total	A	B	C	Total
MCNQ	0	0	0	0	100	100	100	100
MBNQ	0	0	0	0	100	100	100	100
DCNQ	100	100	100	100	100	100	100	100
DBNQ	0	0	0	0	100	100	100	100

**Table 2:** The concentrations of HNQs in source water and finished water of DWTPs in Shanghai.

DWTPs	Samples	Value	Concentration (ng/L)				
			MCNQ	DCNQ	MBNQ	DBNQ	∑HNQs
A Source water	9	Mean	nd	0.18	nd	nd	0.18
		Median		0.18			0.18
		SD		0.01			0.01
		Range		0.14–0.20			0.14–0.20
B Source water	9	Mean	nd	0.21	nd	nd	0.21
		Median		0.22			0.22
		SD		0.01			0.01
		Range		0.19–0.25			0.19–0.25
C Source water	9	Mean	nd	0.50	nd	nd	0.50
		Median		0.50			0.50
		SD		0.01			0.01
		Range		0.49–0.53			0.49–0.53
A Finished water	9	Mean	13.05	2.05	2.10	0.70	17.90
		Median	13.36	2.06	2.03	0.70	18.14
		SD	0.65	0.02	0.09	0.24	0.67
		Range	10.25–14.51	1.80–2.11	1.81–2.43	0.65–0.73	14.90–19.23
B Finished water	9	Mean	11.87	1.01	13.89	1.38	28.17
		Median	13.99	0.60	11.06	1.01	29.23
		SD	1.97	0.30	2.88	0.42	0.96
		Range	5.37–16.07	0.35–1.89	2.05–23.42	0.79–2.38	25.46–30.33
C Finished water	9	Mean	8.7	0.85	1.37	0.76	11.65
		Median	6.32	0.80	1.34	0.76	9.34
		SD	1.89	0.08	0.06	0.23	1.85
		Range	4.12–14.22	0.51–0.94	1.20–1.65	0.73–0.80	7.14–16.87

nd, not detection; ∑HNQs, Total concentration of four HNQs.

In this study, the average concentration of ∑HNQs in source water was 0.30 ng/L, which was higher than the results of Jiang et al. (7). This discrepancy may be explained by the enhanced sensitivity of our analytical method, which achieved lower detection limits. In addition, only DCNQ can be detected in the source water of the DWTPs, probably because DCNQ is an agricultural fungicide that can contaminate water sources through daily agricultural activities.

**Figure 1:**
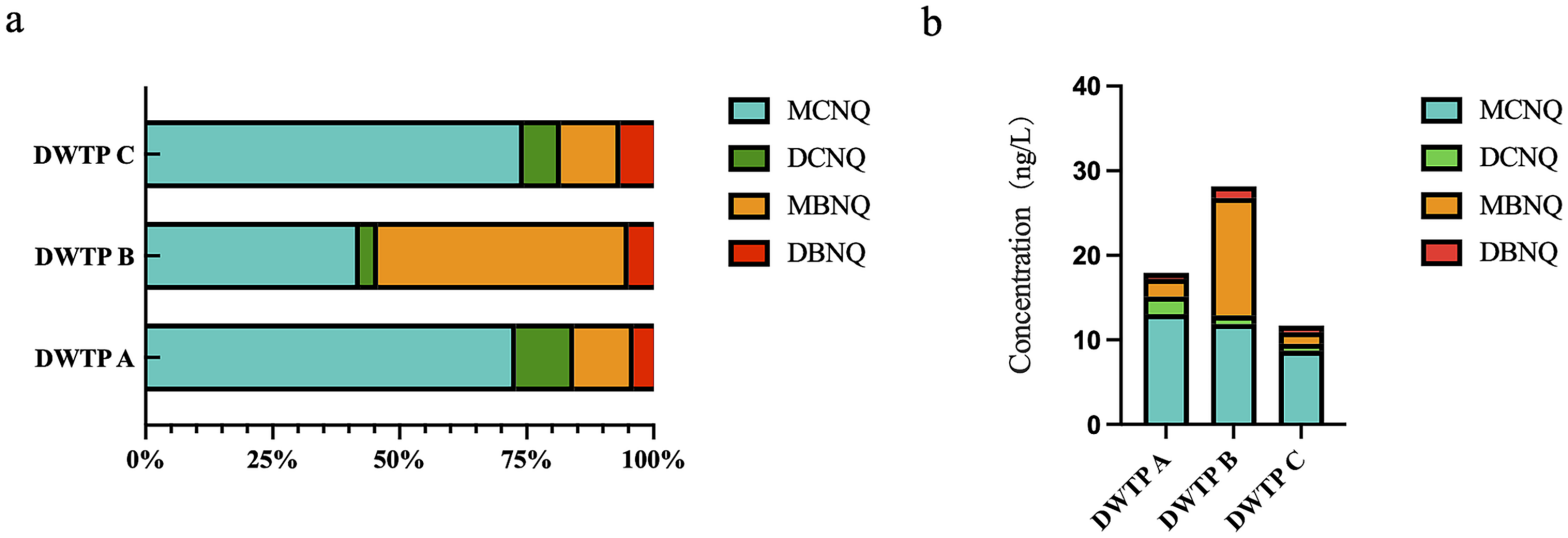
Composition and concentration of HNQs in the finished water of three DWTPs in Shanghai. **(a)** The composition of HNQs in the finished water; **(b)** The concentration of HNQs in the finished water.

The occurrence and distribution of HNQs in finished water from the three DWTPs were presented in Figure 1 and summarized in Tables 1, 2. All four target compounds were detectable in finished water samples from each DWTP. The MCNQ was the most abundant constituent, with average concentrations ranging from 8.7 ng/L to 13.05 ng/L across the three plants, comprising 42.17–74.49% of the total HNQs. Notably, the concentration of MCNQ was significantly higher in DWTP A (13.05 ng/L) than in DWTP C (8.7 ng/L) (*p* = 0.031). The second most prevalent compound was MBNQ, with mean concentrations varying from 1.37 ng/L to13.89 ng/L, accounting for 11.73–49.34% of the total HNQs. Statistical analysis revealed that MBNQ concentrations varied significantly among the DWTPs (*p* < 0.001), with the highest level observed in DWTP B (13.89 ng/L) and the lowest in DWTP C (1.37 ng/L). The average concentrations of DCNQ and DBNQ across the three DWTPs ranged from 0.85 ng/L to 2.05 ng/L and 0.70 ng/L to 1.38 ng/L, respectively. For ∑HNQs, significant differences were also observed among the three DWTPs (*p* < 0.001). DWTP B exhibited the highest ∑HNQs concentration (28.17 ng/L), followed by DWTP A (17.90 ng/L), while DWTP C had the lowest level (11.65 ng/L).

Analysis of finished water from the three DWTPs revealed the presence of all four target HNQs at a detection frequency of 100%, suggesting that these compounds may be formed as DBPs during water treatment. The concentrations of ∑HNQs varied considerably among the DWTPs, with DWTP B exhibiting the highest levels, followed by DWTP A, and DWTP C showing the lowest concentrations. Although all three DWTPs employed advanced treatment processes including ozonation and activated carbon filtration, DWTP C was distinguished by the additional implementation of a submerged ultrafiltration membrane system for source water purification. This membrane treatment step may contribute to the enhanced removal of HNQs precursors or the compounds themselves, thereby resulting in the lower ∑HNQs concentrations observed in finished water from DWTP C. The immersion ultrafiltration membrane can trap suspended solids, colloids, bacteria and other pollutants through the physical screening on the membrane surface with a pore size of 0.002 μm − 0.1 μm (30), and can also remove organic micropollutants (31). The removal rate of organic micropollutants ranged from 63 to 100% (32). This may explain the observations in the present study: although the source water at DWTP C contained higher HNQs levels than those at DWTP A and DWTP B, the application of this advanced treatment process likely contributed to more effective removal of HNQs, resulting in lower concentrations in its finished water.

### 3.3 Cytotoxicity of HNQs to HepG2 cells

**Figure 2:**
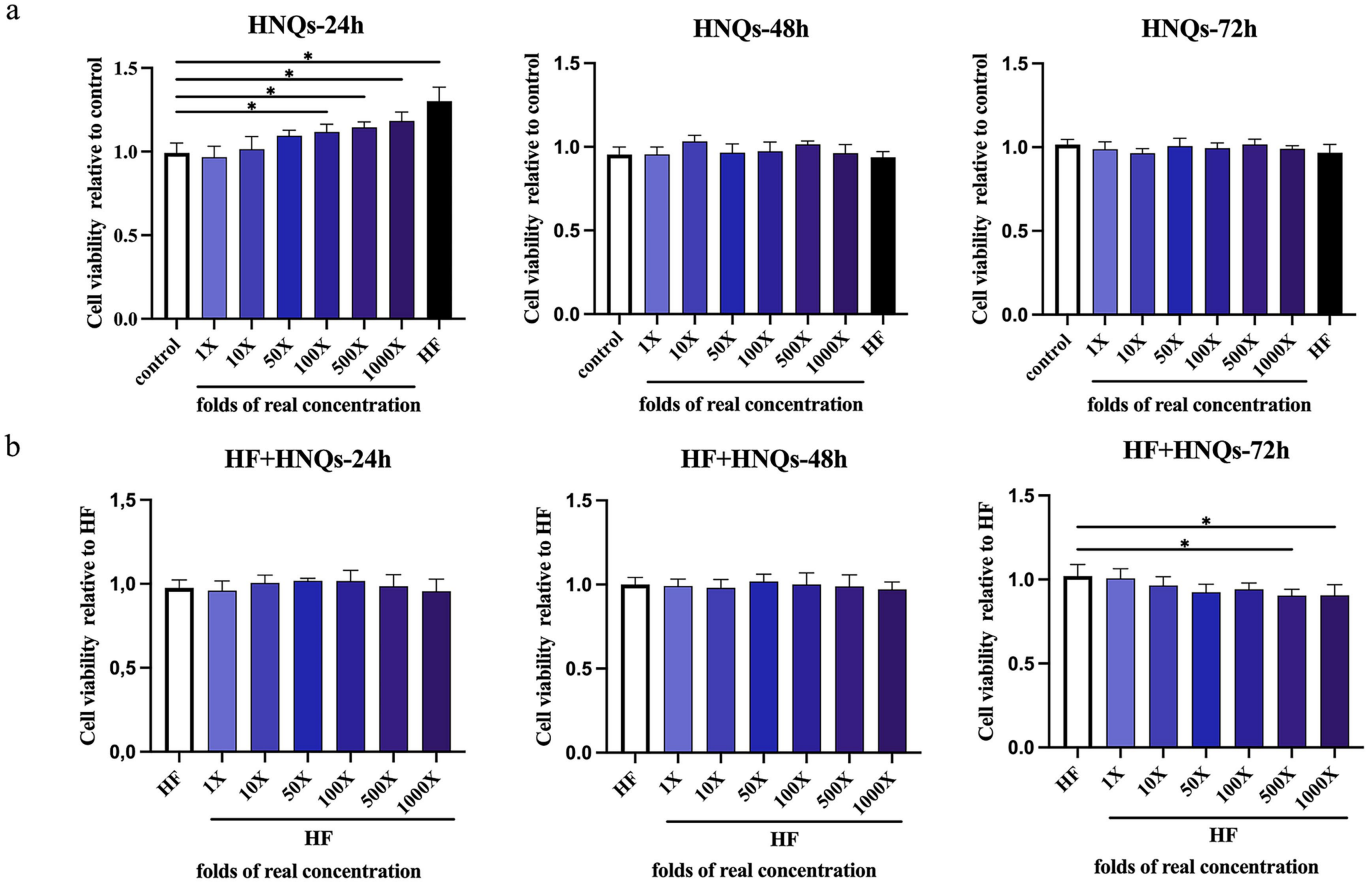
The cytotoxicity of HepG2 cells exposure to HNQs mixture (*n* = 4, Mean ± SD, * *p* < 0.05, Control: 0.5% DMSO, HF: 0.1 mM OA). **(a)** The cytotoxicity of HepG2 cells exposure to HNQs mixture. **(b)** The cytotoxicity of HepG2 cells exposure to HNQs mixture under HF conditions.

The concentrations of HNQs detected in this study were in the ng/L range. However, considering the multiple extrapolation factors typically employed in risk assessment—such as those accounting for interspecies variation, intraspecies sensitivity, and exposure duration—the overall safety factor could readily exceed 1,000 (33). The cytotoxic effects of HNQs on HepG2 cells were assessed following exposure to 1–1,000 times of the mixed concentrations detected in Shanghai drinking water for 24, 48, and 72 h. As shown in Figure 2a, under normal culture conditions, a statistically significant increase in cell viability relative to the control was observed after 24 h of exposure (*p* < 0.001), which may be attributed to the excitotoxicity commonly observed at low doses of toxicants. However, no significant differences in cell viability were detected after 24 h or 72 h of exposure (Figure 2a).

To investigate the combined toxic effects of HNQs and HF conditions on HepG2 cells, we established an HF culture model and evaluated the cytotoxicity of HNQs under this condition (Figure 2b). After 72 h of co-exposure, HNQs at concentrations 500–1,000 times the actual exposure levels significantly reduced cell viability (*p* = 0.003). These results indicate that HF conditions potentiate the cytotoxicity of HNQs in HepG2 cells.

### 3.4 Effect of exposure to HNQs on lipid accumulation in HepG2 cells

The liver serves as the primary metabolic organ in the human body and is consequently a major target for the accumulation and toxicity of exogenous chemicals. Studies have found that exposure to quinones significantly alters hepatic gene expression profiles, disrupting lipid metabolism, biosynthesis, and catabolic processes, with the most pronounced effects on fatty acids metabolism (34). However, there is a paucity of studies investigating the potential hepatotoxic effects of mixed exposure to HNQs.

**Figure 3:**
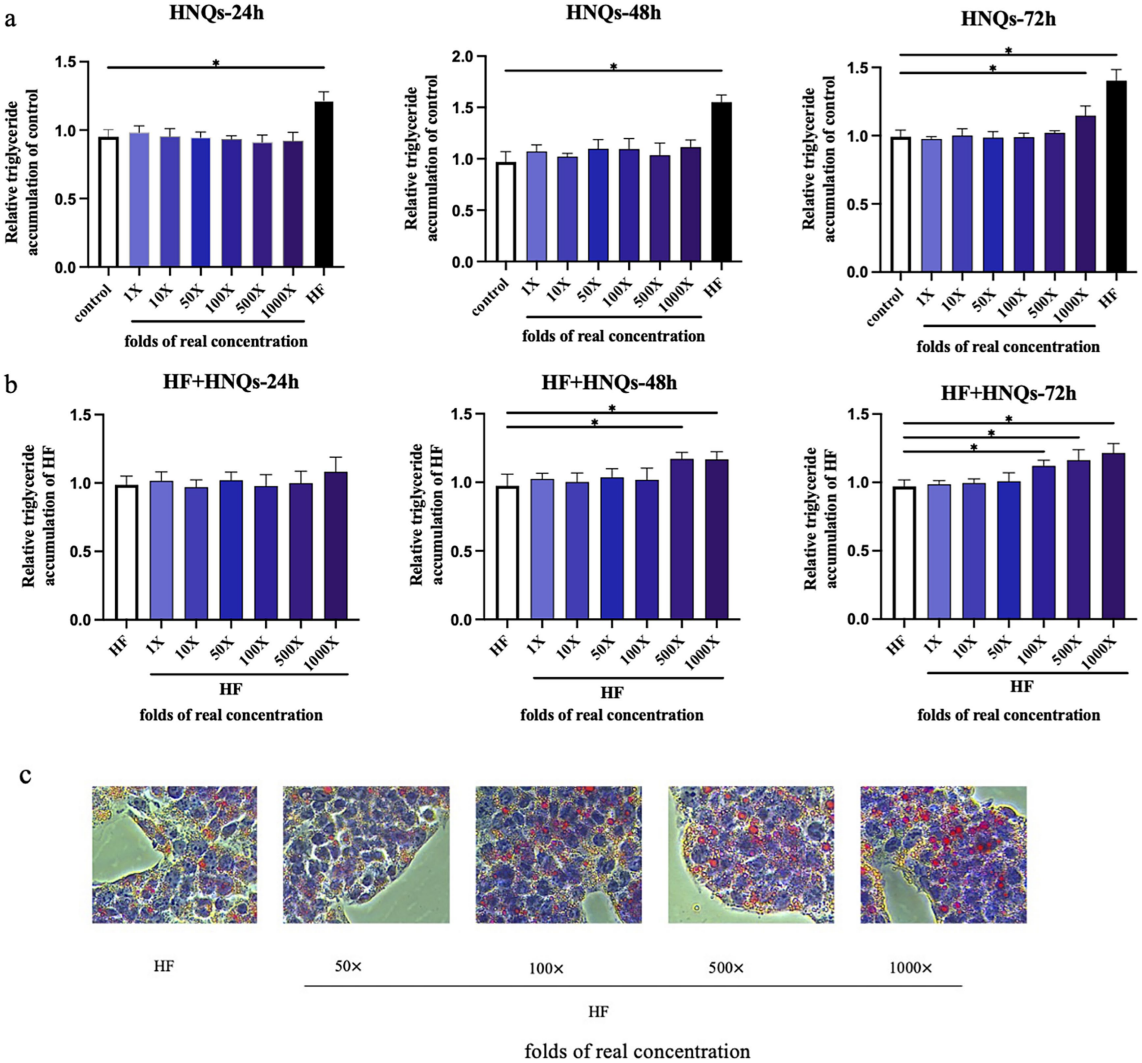
Lipid accumulation effect of HepG2 cells exposure to HNQs mixture under normal and HF conditions (*n* = 4, Mean ± SD, * *p* < 0.05). **(a)** Relative triglyceride accumulation in HepG2 cells exposed to different levels of HNQs concentrations for 24, 48, and 72 h. **(b)** Relative triglyceride accumulation in HepG2 cells exposed to different concentrations of HNQs at 24, 48, and 72 h under HF conditions. **(c)** Oil red O staining results of HepG2 cells exposed to HNQs for 72 h under HF conditions. Control: 0.5% DMSO, HF: 0.1 mM OA.

This study used the HepG2 hepatocyte model to preliminarily investigate the effects of mixed HNQs exposure on hepatocyte lipid accumulation. As illustrated in Figure 3a, under normal conditions, exposure to HNQs for 24 h and 48 h did not induce significant changes in intracellular triglyceride levels. However, following 72 h of exposure, a statistically significant increase in triglyceride accumulation was observed in cells treated with 1,000-fold the actual concentration of HNQs compared to the control group (*p* = 0.001), suggesting that prolonged mixed exposure to HNQs under normal conditions can promote lipid accumulation. Under HF conditions, the lipogenic effect of HNQs was both accelerated and amplified. Significant increases in triglyceride levels were detected after only 48 h of exposure to 500-fold HNQs (*p* = 0.002), and intracellular lipid accumulation exhibited a concentration-dependent increase with increasing HNQs exposure levels (Figures 3b,c). After 48 h of mixed HNQs exposure, the 500- and 1,000-fold concentrations of HNQs induced lipid accumulation in HepG2 cells at 1.20 -fold of the HF control group (*p* = 0.002). After 72 h of exposure, the 100-, 500-, and 1,000-fold treatments resulted in lipid accumulation of 1.15- (*p* = 0.04), 1.20- (*p* < 0.001), and 1.25-fold (*p* < 0.001) of the HF control group, respectively. These findings preliminary demonstrated that HF conditions could sensitize HepG2 cells to HNQs-induced lipid accumulation, resulting in an earlier onset and greater magnitude of response.

Recent studies have linked various environmental pollutants to disrupted lipid metabolism and increased MAFLD risk. For instance, Br-THMs have been associated with collagen deposition, inflammation, and liver necrosis (35), while haloacetamides at low doses promote triglyceride accumulation and alter lipid metabolism pathways in hepatocytes (36). Similarly, zebrafish exposed to HBQs exhibit abnormal lipid peroxidation and fatty acid metabolism (12). Moreover, under HF culture conditions, co-exposure to pollutants such as haloacetaldehyde (33), perf- and polyfluoroalkyl substances, polychlorinated biphenyls (37), and vinyl chloride (38), has been shown to significantly aggravate hepatic lipid accumulation. Given the marked effects of HNQs on lipid metabolism in hepatocytes, further research is warranted to characterize the occurrence and biological effects of unidentified emerging DBPs in drinking water with related structures and higher molecular weight, and further investigation into lipid-DBPs interactions is also needed.

### 3.5 Effect of exposure to HNQs on oxidative stress in HepG2 cells

Previous studies have demonstrated that the adverse effects of quinones are predominantly attributable to oxidative stress (39, 40). Likewise, HBQs, which are structurally similar to HNQs, have been found to lower glutathione reserves, stimulate lipid peroxidation, and interfere with fatty acid metabolism (17), implying that HNQs could elicit hepatocyte injury via analogous routes.

**Figure 4:**
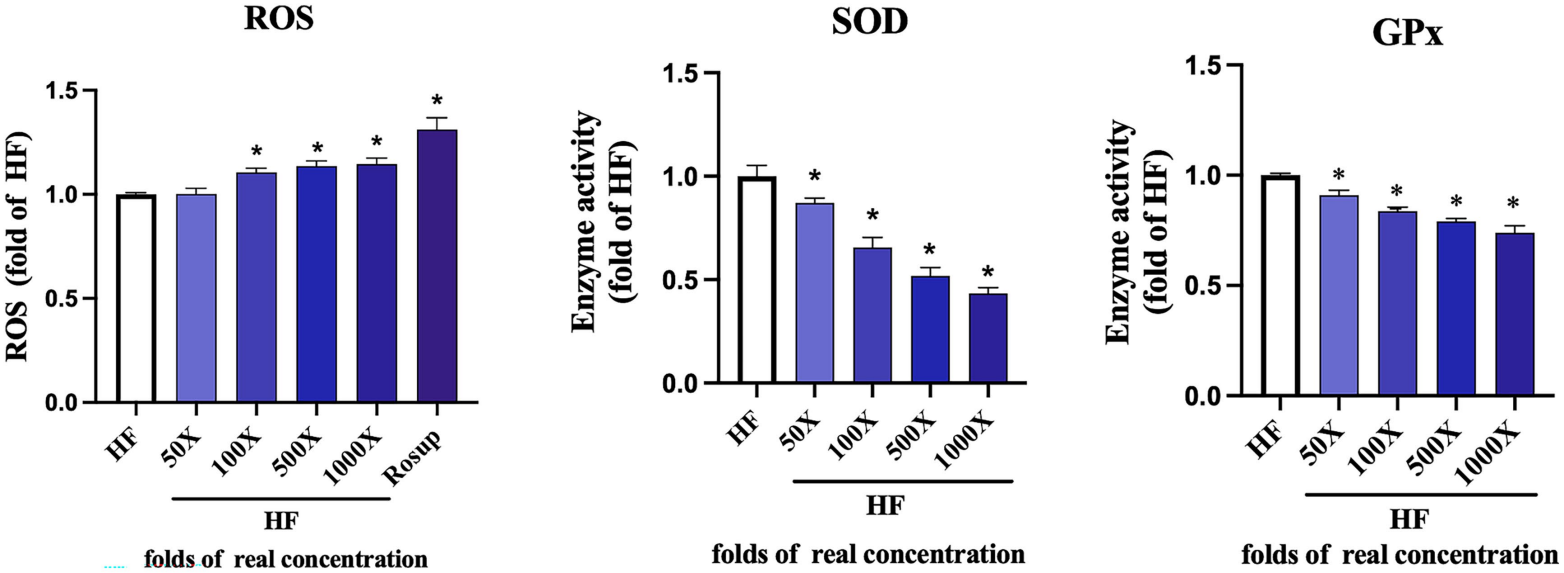
ROS content, SOD and GPx enzyme activities of HepG2 cells exposed to HNQs mixture under HF conditions. (Mean ± SD, *n* = 4, *p < 0.05, HF: 0.1 mM OA, Rosup: reactive oxygen positive control).

Accordingly, we investigated the effects of exposure to environmentally relevant concentrations of HNQs on oxidative stress markers in HepG2 cells under HF conditions (Figure 4). The results showed that, under HF conditions, exposure to HNQs led to a gradual increase in intracellular reactive oxygen species (ROS) levels compared to the control group (*p* < 0.05). Concurrently, the activities of antioxidant enzymes, including superoxide dismutase (SOD) and glutathione peroxidase (GPx), decreased progressively with increasing HNQs concentrations (*p* < 0.05).

Within the cellular antioxidant defense system, SOD and GPx play indispensable and complementary roles. Together, these enzymes constitute a primary frontline against oxidative injury, effectively scavenging ROS and eliminating harmful lipid peroxidation products, thereby protecting cells from oxidative damage. This study demonstated that mixed exposure to HNQs may cause lipid metabolism abnormalities through oxidative stress, potentially contributing to the development of MAFLD. Previous investigations have demonstrated that exposure to HBQs disrupts this delicate redox balance in HepG2 cells, leading to enhanced ROS production (41) and a concomitant reduction in SOD activity, indicative of oxidative stress (34). HBQs exposure also induces significant hepatotoxicity in mice (21, 42), as evidenced by markedly elevated serum alanine aminotransferase and aspartate aminotransferase activities, along with histopathological changes including hepatocyte vacuolization, nuclear pyknosis, and inflammatory cell infiltration (43). Accumulation of lipid peroxidation products amplifies cell damage and sustains oxidative stress, potentially serving as a “second hit” that triggers inflammatory responses and cellular injury—a key mechanism in the progression of MAFLD (44).

The exposure doses in this study were set at 50- to 1,000-fold multiples of the mixed HNQ concentrations detected in Shanghai drinking water. Given the direct cell contact under *in vitro* conditions, the effective cellular exposure likely far exceeds the concentrations that would reach the liver following oral ingestion. However, the incidence and severity of lipid dysfunction and hepatotoxicity in the human population are steadily increasing, underscoring the value of such high-dose regimens for elucidating the mode of action of HNQs. *In vitro-in vivo* extrapolation of dosimetry is crucial for assessing expected tissue exposures in the intestinal epithelium and liver, particularly for chronic, intermittent exposure to emerging DBPs via drinking water. Future studies can integrate our current findings with pharmacokinetic modeling to better predict post-ingestion HNQ distribution to the gut and liver and clarify their potential contributions to human diseases such as MAFLD. Although extrapolating from higher-dose *in vitro* data to real-world low-dose scenarios presents challenges, these experimental approaches offer the distinct advantage of revealing mechanistic insights into compound activity.

### 3.6 Effects of exposure to HNQs on the expression of lipid metabolism-related genes and autophagy-related genes in HepG2 cells

**Figure 5:**
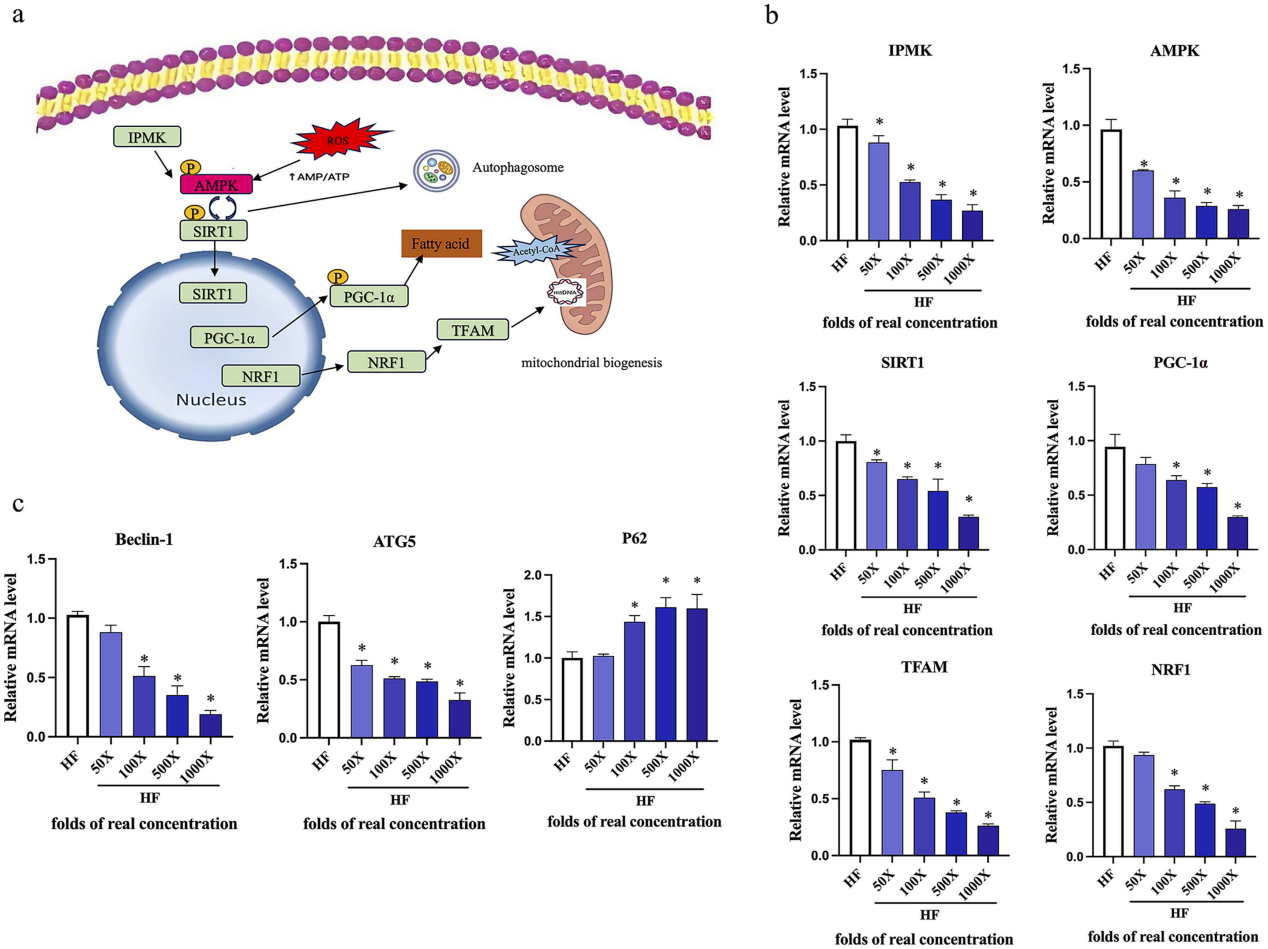
Effects of exposure to HNQs mixture on the expression of genes related to lipid metabolism in HepG2 cells (Mean ± SD, *n* = 3, * *p* < 0.05, HF: 0.1 mM OA). **(a)** Schematic for pathways of related genes in cells. **(b)** Effect on the expression of mRNA of lipid metabolism-related genes in HepG2 cells. **(c)** Effect on the expression of mRNA of autophagy-related genes in HepG2 cells.

Excessive ROS accumulation, when exceeding the neutralization capacity of antioxidant enzymes, can trigger severe downstream effects, including mitochondrial dysfunction, impaired *β*-oxidation of free fatty acids, and the initiation of lipid peroxidation cascades (45). Activation of the *IPMK/AMPK/SIRT1/PGC-1α* signaling pathway has been shown to counteract oxidative stress and inflammation (Figure 5a), thereby playing a pivotal role in antioxidant defense (23, 24). Within this pathway, *AMPK* and *SIRT1* form a positive feedback loop, co-regulating *PGC-1α*, a master regulator of mitochondrial energy metabolism. Once activated, *PGC-1α* upregulates nuclear respiratory factor 1 (*NRF1*), which in turn activates mitochondrial transcription factor A (*TFAM*), promoting mitochondrial DNA replication and transcription. This cascade enhances mitochondrial function, alleviates oxidative stress damage, sustains normal fatty acid metabolism and gluconeogenesis, and ultimately attenuates hepatic lipid accumulation (46, 47).

Based on the oxidative stress findings, we further investigated the effects of mixed HNQs exposure on the expression of lipid metabolism-related genes in hepatocytes under the HF culture conditions. As shown in Figure 5b, the expression levels of inositol polyphosphate multikinase (*IPMK*), *AMPK*, *SIRT1*, and *PGC-1α* exhibited a concentration-dependent decrease with increasing HNQs concentrations (*p* < 0.05). Consistently, the present study found that after 72 h of exposure to HNQs, the expression levels of *NRF1* and *TFAM* were progressively downregulated in HepG2 cells with increasing HNQs dose. Hepatic fat accumulation is closely associated with dysregulated lipid metabolism and disruption of autophagic pathways. In the present study, exposure to HNQs resulted in a dose-dependent decrease in the expression of autophagy-related genes *Beclin-1* and *Atg5* in HepG2 cells, accompanied by a concurrent increase in the expression of sequestosome-1 (*P62*) (*p* < 0.05; Figure 5c).

Previous studies have demonstrated that HBQs suppress *AMPK* and *SIRT1* expression by altering the intracellular AMP/ATP ratio (48, 49), leading to reduced expression of *PGC-1α* in skeletal muscle and inducing lipid accumulation. In HepG2 cells, *SIRT1* upregulation has been reported to repress lipogenic gene expression upon FFA stimulation (50). *Beclin-1* and *Atg5* are key regulators of autophagosome formation, and *AMPK* activation has been shown to directly activate *Beclin-1*, thereby promoting autophagy and reducing lipid accumulation and steatosis (51). Studies have found that oxidative stress levels increase in hepatitis patients, with a declining trend in serum *Beclin-1* levels, correlating with MAFLD development (52). These findings suggest that HNQs exposure may induce hepatocyte mitochondrial dysfunction by disrupting the *IPMK/AMPK/SIRT1/PGC-1α* signaling axis, leading to abnormal lipid metabolism in hepatocytes. Although protein-levels validation was not performed in this study, the functional roles of the genes encoding these proteins have been established in similar models (53, 54). Lipophilic pollutants accumulated in adipose tissue in obesity are released into the bloodstream during weight fluctuations and redistributed to the liver with blood circulation, inhibiting the *AMPK* pathway (55) and hindering mitochondrial autophagy, leading to energy metabolism disorders, which may be the reason for the further increase in lipid levels under HF conditions.

## 4 Conclusion

This study employed the LLE-HPLC-MS/MS to characterize the contamination levels and distribution profiles of HNQs in drinking water, and utilized the HepG2 cell model to investigate the effects of HNQs exposure on hepatic lipid metabolism. We demonstrate that HNQs in drinking water represent a risk factor for MAFLD. HNQs induce excessive oxidative stress in hepatocytes, which may compromise mitochondrial function, disrupt redox homeostasis and interfere with normal lipid metabolism. Given these findings, further investigation into the occurrence and biological effects of structurally analogous, higher-molecular-weight DBPs in drinking water is warranted, with important implications for water quality surveillance and public health protection. Furthermore, to better understand the toxicological consequences of low-dose mixed exposure, future studies should simulate actual exposure scenarios and investigate the effects of long-term co-exposure to HNQs and HF at environmentally relevant levels on hepatocyte lipogenesis and lipotoxicity, thereby clarifying the health risks posed to the general population, particularly high-risk subgroups.

## Data Availability

The datasets presented in this study can be found in online repositories. The names of the repository/repositories and accession number(s) can be found in the article/Supplementary material.

## References

[1] Forster ALB Wiskur SL Richardson SD. Formation of eight classes of Dbps from chlorine, chloramine, and ozone: mechanisms and formation pathways. Environ Sci Technol. (2025) 59:15594–611. doi: 10.1021/acs.est.5c02208, 40689434

[2] Krasner SW Westerhoff P Chen B Rittmann BE Amy G. Occurrence of disinfection byproducts in United States wastewater treatment plant effluents. Environ Sci Technol. (2009) 43:8320–5. doi: 10.1021/es901611m, 19924963

[3] Wang J Shen J Ye D Yan X Zhang Y Yang W . Disinfection technology of hospital wastes and wastewater: suggestions for disinfection strategy during coronavirus disease 2019 (Covid-19) pandemic in China. Environ Pollut. (2020) 262:114665. doi: 10.1016/j.envpol.2020.114665, 32443202 PMC7194566

[4] Wang L Zhang X Chen S Meng F Zhang D Liu Y . Spatial variation of dissolved organic nitrogen in Wuhan surface waters: correlation with the occurrence of disinfection byproducts during the Covid-19 pandemic. Water Res. (2021) 198:117138. doi: 10.1016/j.watres.2021.117138, 33895589 PMC8036133

[5] Richardson SD Plewa MJ Wagner ED Schoeny R Demarini DM. Occurrence, genotoxicity, and carcinogenicity of regulated and emerging disinfection by-products in drinking water: a review and roadmap for research. Mutat Res. (2007) 636:178–242. doi: 10.1016/j.mrrev.2007.09.001, 17980649

[6] Zhang D Chu WH Yu Y Krasner SW Pan Y Shi J . Occurrence and stability of chlorophenylacetonitriles: a new class of nitrogenous aromatic DBPs in chlorinated and chloraminated drinking waters. Environ Sci Technol Lett. (2018) 5:394–9. doi: 10.1021/acs.estlett.8b00220

[7] Jiang HC Kaw HY Zhu LZ Wang W. Halonaphthoquinones: a group of emerging disinfection byproducts of high toxicity in drinking water. Water Res. (2022) 217:421. doi: 10.1016/j.watres.2022.118421, 35429882

[8] Yang L Zhang X Xing WL Zhou QY Zhang LL Wu Q . Yearly variation in characteristics and health risk of polycyclic aromatic hydrocarbons and nitro-PAHs in urban Shanghai from 2010-2018. J Environ Sci (China). (2021) 99:72–9. doi: 10.1016/j.jes.2020.06.017, 33183718

[9] Sola MCR Santos AG Nascimento MM Da Rocha GO De Andrade JB. Occurrence, sources, and risk assessment of unconventional polycyclic aromatic compounds in marine sediments from sandy beach intertidal zones. Sci Total Environ. (2022) 810:152019. doi: 10.1016/j.scitotenv.2021.152019, 34856251

[10] Idowu O Carbery M O'connor W Thavamani P. Speciation and source apportionment of polycyclic aromatic compounds (PACs) in sediments of the largest salt water lake of Australia. Chemosphere. (2020) 246:125779. doi: 10.1016/j.chemosphere.2019.125779 31927372

[11] Said TO El Agroudy NA. Assessment of PAHs in water and fish tissues from great bitter and El Temsah lakes, Suez Canal, as chemical markers of pollution sources. Chem Ecol. (2006) 22:159–73. doi: 10.1080/02757540500526476

[12] Wang CH Zhou SL Wu SH Song J Shi YX Li BJ . Surface water polycyclic aromatic hydrocarbons (PAH) in urban areas of Nanjing, China. Water Sci Technol. (2017) 76:2150–7. doi: 10.2166/wst.2017.387, 29068344

[13] Gustafson KE Dickhut RM. Distribution of polycyclic aromatic hydrocarbons in southern Chesapeake Bay surface water: evaluation of three methods for determining freely dissolved water concentrations. Environ Toxicol Chem. (1997) 16:452–61. doi: 10.1002/etc.5620160310

[14] Hayakawa K Makino F Yasuma M Yoshida S Chondo Y Toriba A . Polycyclic aromatic hydrocarbons in surface water of the southeastern Japan Sea. Chem Pharm Bull. (2016) 64:625–31. doi: 10.1248/cpb.c16-00063, 27250797

[15] Jena AB Samal RR Kumari K Pradhan J Chainy GBN Subudhi U . The benzene metabolite p-benzoquinone inhibits the catalytic activity of bovine liver catalase: a biophysical study. Int J Biol Macromol. (2021) 167:871–80. doi: 10.1016/j.ijbiomac.2020.11.044, 33181220

[16] Pan H Zhai G Jing Q Fan Y Fang C Shi F. Two-step metabolic activation to ortho-benzoquinone intermediate and its role in 2,3,5,4′-tetrahydroxystilbene-2-O-β-D-glucoside-induced liver injury in mice. Drug Metab Dispos. (2025) 53:100047. doi: 10.1016/j.dmd.2025.100047, 40037093

[17] Li JH Moe B Vemula S Wang W Li XF. Emerging disinfection byproducts, Halobenzoquinones: effects of isomeric structure and halogen substitution on cytotoxicity, formation of reactive oxygen species, and genotoxicity. Environ Sci Technol. (2016) 50:6744–52. doi: 10.1021/acs.est.5b05585, 26812484

[18] Tu N Liu H Li W Yao S Liu J Guo Z . Quantitative structure-toxicity relationships of halobenzoquinone isomers on Dna reactivity and genotoxicity. Chemosphere. (2022) 309:136763. doi: 10.1016/j.chemosphere.2022.136763, 36209857

[19] Wu H Long KL Sha YJ Lu D Xia Y Mo Y . Occurrence and toxicity of halobenzoquinones as drinking water disinfection byproducts. Sci Total Environ. (2021) 770:277. doi: 10.1016/j.scitotenv.2021.145277, 33515874

[20] Zheng ZY Ni HG. Predicted no-effect concentration for eight Pahs and their ecological risks in seven major river systems of China. Sci Total Environ. (2024) 906:167590. doi: 10.1016/j.scitotenv.2023.167590, 37802352

[21] Siraki AG Chan TS O'brien PJ. Application of quantitative structure-toxicity relationships for the comparison of the cytotoxicity of 14 p-benzoquinone congeners in primary cultured rat hepatocytes versus Pc12 cells. Toxicol Sci. (2004) 81:148–59. doi: 10.1093/toxsci/kfh182, 15178806

[22] Xu DM Hu LH Xia XM Song JB Li LR Song EQ . Tetrachlorobenzoquinone induces acute liver injury, up-regulates Ho-1 and Nqo1 expression in mice model: the protective role of chlorogenic acid. Environ Toxicol Pharmacol. (2014) 37:1212–20. doi: 10.1016/j.etap.2014.04.022, 24816176

[23] Guha P Snyder SH. Noncatalytic functions of Ipmk are essential for activation of autophagy and liver regeneration. Autophagy. (2019) 15:1473–4. doi: 10.1080/15548627.2019.1615305, 31066329 PMC6613895

[24] Jung IR Ahima RS Kim SF. Ipmk modulates Ffa-induced insulin resistance in primary mouse hepatocytes. bioRxiv. (2023) 2023:310. doi: 10.1101/2023.04.26.538310, 37795573

[25] Lee MR Yang HJ Park KI Ma JY. *Lycopus lucidus* Turcz. Ex Benth. Attenuates free fatty acid-induced steatosis in HepG2 cells and non-alcoholic fatty liver disease in high-fat diet-induced obese mice. Phytomedicine. (2019) 55:14–22. doi: 10.1016/j.phymed.2018.07.008, 30668424

[26] Abeyratne-Perera HK Basu S Chandran PL. Shells of compacted DNA as nanocontainers transporting proteins in multiplexed delivery. Mater Sci Eng C Mater Biol Appl. (2021) 127:112184. doi: 10.1016/j.msec.2021.112184, 34225845

[27] Liew CS Li X Lee HK. Miniscale liquid-liquid extraction coupled with full evaporation dynamic headspace extraction for the gas chromatography/mass spectrometric analysis of polycyclic aromatic hydrocarbons with 4000-to-14 000-fold enrichment. Anal Chem. (2016) 88:9095–102. doi: 10.1021/acs.analchem.6b02056, 27535573

[28] Antunes P Viana P Vinhas T Capelo JL Rivera J Gaspar EM. Optimization of pressurized liquid extraction (Ple) of dioxin-furans and dioxin-like Pcbs from environmental samples. Talanta. (2008) 75:916–25. doi: 10.1016/j.talanta.2007.12.042, 18585164

[29] Wenling L Lanhui L Liujun C Dongmei W Shiyue Z. Improvement of method for determing dibutyl phthalate in surface water by high performance liquid chromatography. Yunnan Huagong. (2023) 50:75–8.

[30] Comerton AM Andrews RC Bagley DM Yang P. Membrane adsorption of endocrine disrupting compounds and pharmaceutically active compounds. J Membr Sci. (2007) 303:267–77. doi: 10.1016/j.memsci.2007.07.025

[31] Xia SJ Li X Zhang QL Xu B Li GB. Ultrafiltration of surface water with coagulation pretreatment by streaming current control. Desalination. (2007) 204:351–8. doi: 10.1016/j.desal.2006.03.544

[32] Mroczko D Zimoch I Kryczka M. Effectiveness of ultra-, nanofiltration and selected photolytic processes in removing of selected priority substances from water. Desalin Water Treat. (2023) 314:59–69. doi: 10.5004/dwt.2023.30066

[33] Yang L Jiang Z Yang L Zheng W Chen Y Qu F . Disinfection byproducts of Haloacetaldehydes disrupt hepatic lipid metabolism and induce lipotoxicity in high-fat culture conditions. Environ Sci Technol. (2024) 58:12356–67. doi: 10.1021/acs.est.3c11009, 38953388

[34] Wang YY Wang FB Li LL Zhang L Song MY Jiang GB. Comprehensive toxicological assessment of Halobenzoquinones in drinking water at environmentally relevant concentration. Environ Sci Technol. (2024) 58:9125–34. doi: 10.1021/acs.est.4c03308, 38743861

[35] Das S Kumar A Seth RK Tokar EJ Kadiiska MB Waalkes MP . Proinflammatory adipokine leptin mediates disinfection byproduct bromodichloromethane-induced early steatohepatitic injury in obesity. Toxicol Appl Pharmacol. (2013) 269:297–306. doi: 10.1016/j.taap.2013.02.003, 23438451 PMC3654077

[36] Jiang Z Yang L Liu Q Qiu M Chen Y Qu F . Haloacetamides disinfection by-products, a potential risk factor for nonalcoholic fatty liver disease. Water Res. (2024) 261:122008. doi: 10.1016/j.watres.2024.122008, 38944971

[37] Lang AL Chen L Poff GD Ding WX Barnett RA Arteel GE . Vinyl chloride dysregulates metabolic homeostasis and enhances diet-induced liver injury in mice. Hepatol Commun. (2018) 2:270–84. doi: 10.1002/hep4.1151, 29507902 PMC5831023

[38] Wahlang B Song M Beier JI Cameron Falkner K Al-Eryani L Clair HB . Evaluation of Aroclor 1260 exposure in a mouse model of diet-induced obesity and non-alcoholic fatty liver disease. Toxicol Appl Pharmacol. (2014) 279:380–90. doi: 10.1016/j.taap.2014.06.019, 24998970 PMC4225625

[39] Jia K Sun J Du Q Qu Y Han J Liu H . Mass spectrometry imaging unveils the metabolic effect of 6ppd-quinone in exposed mice. Environ Sci Technol. (2025) 59:4282–91. doi: 10.1021/acs.est.4c11156, 40000248

[40] Krylova NG Kulahava TA Cheschevik VT Dremza IK Semenkova GN Zavodnik IB. Redox regulation of mitochondrial functional activity by quinones. Physiol Int. (2016) 103:439–58. doi: 10.1556/2060.103.2016.4.4, 28229632

[41] Hung S Mohan A Reckhow DA Godri Pollitt KJ. Assessment of the in vitro toxicity of the disinfection byproduct 2,6-dichloro-1,4-benzoquinone and its transformed derivatives. Chemosphere. (2019) 234:902–8. doi: 10.1016/j.chemosphere.2019.06.086, 31519098

[42] Zhou M Li J Du M Wang J Kaw HY Zhu L . Methoxylated modification of glutathione-mediated metabolism of halobenzoquinones in vivo and in vitro. Environ Sci Technol. (2023) 57:3581–9. doi: 10.1021/acs.est.2c06765, 36802564

[43] Xu Z. Liver Damage of mice Induced by the Joint Exposure of Waterdisinfection Byproducts Halobenzoquinone and N-Nitrosamine. Changchun: Jilin University (2024).

[44] Xiao M Zhong H Xia L Tao Y Yin H. Pathophysiology of mitochondrial lipid oxidation: role of 4-hydroxynonenal (4-Hne) and other bioactive lipids in mitochondria. Free Radic Biol Med. (2017) 111:316–27. doi: 10.1016/j.freeradbiomed.2017.04.363, 28456642

[45] Zhang Y Sano M Shinmura K Tamaki K Katsumata Y Matsuhashi T . 4-hydroxy-2-nonenal protects against cardiac ischemia-reperfusion injury via the Nrf2-dependent pathway. J Mol Cell Cardiol. (2010) 49:576–86. doi: 10.1016/j.yjmcc.2010.05.011, 20685357

[46] Ren R Wang Z Wu M Wang H. Emerging roles of Sirt1 in alcoholic liver disease. Int J Biol Sci. (2020) 16:3174–83. doi: 10.7150/ijbs.49535, 33162823 PMC7645991

[47] Tang BL. Sirt1 and the mitochondria. Mol Cells. (2016) 39:87–95. doi: 10.14348/molcells.2016.2318, 26831453 PMC4757807

[48] Lu F Zhang Q Zhang M Sun S Yang X Yan H. Blocking exosomal secretion aggravates 1,4-benzoquinone-induced mitochondrial fission activated by the Ampk/Mff/Drp1 pathway in Hl-60 cells. J Appl Toxicol. (2022) 42:1618–27. doi: 10.1002/jat.4328, 35383983

[49] Son HJ Jang YJ Jung CH Ahn J Ha TY. 2,6-Dimethoxy-1,4-benzoquinone inhibits 3T3-L1 adipocyte differentiation via regulation of Ampk and mtorc1. Planta Med. (2019) 85:210–6. doi: 10.1055/a-0725-8334, 30199902

[50] Long JK Dai W Zheng YW Zhao SP. miR-122 promotes hepatic lipogenesis via inhibiting the Lkb1/Ampk pathway by targeting Sirt1 in non-alcoholic fatty liver disease. Mol Med. (2019) 25:26. doi: 10.1186/s10020-019-0085-2, 31195981 PMC6567918

[51] Wu W Wang X Sun Y Berleth N Deitersen J Schlutermann D . Tnf-induced necroptosis initiates early autophagy events via Ripk3-dependent Ampk activation, but inhibits late autophagy. Autophagy. (2021) 17:3992–4009. doi: 10.1080/15548627.2021.1899667, 33779513 PMC8726653

[52] Bagheri Lankarani K Sadidoost A Fattahi M Amirizadeh Fard S Mokarram P. The potential role of autophagy in progression of liver fibrosis in chronic hepatitis B patients receiving antiviral treatment: a brief report. Iran J Med Sci. (2024) 49:196–200. doi: 10.30476/ijms.2023.96588.2813, 38584654 PMC10997855

[53] Lee GH Peng C Lee HY Park SA Hoang TH Kim JH . D-allulose ameliorates adiposity through the Ampk-Sirt1-Pgc-1α pathway in Hfd-induced Sd rats. Food Nutr Res. (2021) 65:7803. doi: 10.29219/fnr.v65.7803, 35221861 PMC8829832

[54] Zhuge A Li S Han S Yuan Y Shen J Wu W . *Akkermansia muciniphila*-derived acetate activates the hepatic Ampk/Sirt1/Pgc-1α axis to alleviate ferroptosis in metabolic-associated fatty liver disease. Acta Pharm Sin B. (2025) 15:151–67. doi: 10.1016/j.apsb.2024.10.010, 40041901 PMC11873632

[55] Gao Y Zhang W Zeng LQ Bai H Li J Zhou J . Exercise and dietary intervention ameliorate high-fat diet-induced Nafld and liver aging by inducing lipophagy. Redox Biol. (2020) 36:101635. doi: 10.1016/j.redox.2020.101635, 32863214 PMC7365984

