# Supplementary material for: Occurrence of halonaphthoquinone disinfection byproducts in Shanghai’s drinking water and the hepatotoxic effects on lipid accumulation
Source: Front Public Health. 2026 Jul 7;14:1831866. doi: 10.3389/fpubh.2026.1831866 (PMC13385284; doi:10.3389/fpubh.2026.1831866)
Supplement: Supplementary file 1 [file Data_Sheet_1.docx]

**Supporting Information**

**Occurrence of halonaphthoquinone disinfection byproducts in Shanghai’s drinking water and the hepatotoxic effects on lipid accumulation**

**Text S1 Reagents**

2-bromo-1,4-naphthoquinone (MBNQ,98%), 2,3-dibromo-1,4-naphthoquinone (DBNQ, ≥97%) and n-hexane (HPLC grade) were purchased from the Sigma-Aldrich (St. Louis, USA). 2,3-dichloro-1,4-naphthoquinone (DCNQ, ≥98%), 2-chloro-1,4-naphthoquinone (MBNQ，≥98%), formic acid (≥99%), sodium oleate (OA), sulfuric acid (75%) and sodium hydroxide solution (12.5mol/L) were purchased from Aladdin (Shanghai, China). Methanol (HPLC grade), isopropyl alcohol (≥99%), Oil red O (≥75%) and sodium sulfate anhydrous were purchased from Merck (Darmstadt, Germany). fetal bovine serum (FBS) was purchased from Yeasen (Shanghai, China). Dulbecco's modified Eagle's medium (DMEM) and trypsin-EDTA were purchased from Invitrogen (Carlsbad, CA, USA). 4% paraformaldehyde and NucBlueTM Live ReadyProbesTM kit were purchased from Thermo Fisher Scientific (Braunschweig, Germany). AdipoRed was purchased from Lonza (Walkersville, MD, USA). Hematoxylin staining solution, dimethyl sulfoxide (DMSO), Total Superoxide Dismutase Assay Kit with WST-8, Reactive Oxygen Species Assay Kit, Total Glutathione Peroxidase Assay Kit with NADPH, BCA Protein Assay Kit and RNAeasy™ Animal RNA Isolation Kit with Spin Column were purchased from Beyotime (Shanghai, China). HiScript III RT SuperMix for qPCR (+gDNA wiper) and SupRealQ Purple Universal SYBR qPCR Master Mix (U+) were purchased from Vazyme (Nanjing, China).

**TextS2 Sample preparation**

The liquid-liquid extraction procedure for the isolation of HNQs from water samples was carried out according to the following protocol:

(1) A 500 mL aliquot of the water sample was introduced into a separatory funnel. The funnel was sealed and shaken vigorously to ensure thorough mixing, then allowed to stand undisturbed for 30 min.

(2) The target compounds were then extracted using a specified volume of solvent n-hexane. The mixture was vigorously shaken, and after phase separation, the organic phase was collected in a 15 mL quartz test tube. This extraction step was repeated as necessary.

(3) The combined organic extract was dehydrated by the addition of approximately 0.5 g of anhydrous sodium sulfate to remove any residual water. The dried extract was then carefully transferred to a 15 mL amber sample tube.

(4) The extract was concentrated under a gentle stream of high-purity nitrogen in a thermostatically controlled water bath set at 50 °C until near dryness.

The concentrated extract was reconstituted in 1 mL of methanol containing 0.25% formic acid. The resulting solution was then filtered through a 0.22 μm membrane filter into a brown autosampler vial and subsequently analyzed by HPLC-MS/MS.

To ensure optimal recovery of the four HNQs compounds from water samples, key parameters of the liquid-liquid extraction pretreatment were systematically optimized. The volume of extraction solvent n-hexane was optimized by testing four different volumes (5, 10, 15, and 20 mL) for their efficiency in enriching HNQs. Following selection of the optimal solvent volume, the influence of sample pH on extraction efficiency was investigated. The pH of water samples was adjusted to various values using sulfuric acid or sodium hydroxide prior to extraction, and the resulting recoveries of HNQs were compared to identify the pH condition that maximized extraction performance.

**TextS3 HPLC-MS/MS**

The optimized gradient elution program was completed in 18 minutes at a flow rate of 0.25 mL/min. The mobile phase consisted of (A) ultrapure water and (B) methanol. The flow rate was 0.25 mL/min, and the following instrument parameters were used: 10 μL injection volume and 40 °C column temperature. The gradient elution procedure is as follows: solvent A changed linearly from 90% to 40% in the 3 minutes, and then decreased from 40% to 10% in the following 10 minutes, returned to 90% in the next 0.1 minutes, and finally maintained for 4.9 minutes to re-equilibrate the column.

Chromatographic separation and mass spectrometric detection of the target analytes HNQs were carried out using a Shimadzu High-Performance Liquid Chromatography system (LC-20A) interfaced with an AB SCIEX QTRAP 5500 triple quadrupole ion-trap tandem mass spectrometer. The analytical column employed was a BEH C18 column (2.1 mm × 100 mm × 1.7 μm), which provided efficient separation of the four HNQs compounds. The chromatographic separation was carried out under the following conditions: flow rate of 0.25 mL/min, injection volume of 10 μL, and column temperature maintained at 40 °C. The mobile phase consisted of ultrapure water (solvent A) and methanol (solvent B), delivered according to the following optimized gradient elution profile: from 0 to 3 min, the proportion of solvent A was linearly decreased from 90% to 40%; from 3 to 13 min, solvent A was further reduced linearly from 40% to 10%; at 13.1 min, solvent A was returned to 90% within 0.1 min; and from 13.1 to 18 min, the composition was held at 90% A to re-equilibrate the column prior to the next injection. The total run time was 18 min.

The MS parameters were as follows: electron spray ionization (ESI); ion source temperature, 700°C; ion spray voltage, -4500V; entrance potential, -10V; curtain gas, 30 arbitrary units; collision gas, medium; gas I, 50 arbitrary units; gas II, 50 arbitrary units. The sample instrument analysis was described in detail in Table S1 and Figure S1. Mass spectrometric detection was carried out using an electrospray ionization (ESI) source operating in negative ion mode. The optimized source and gas parameters were as follows: ion spray voltage, -4500 V; ion source temperature, 700 °C; entrance potential, -10 V; curtain gas, 30 units; collision gas, medium. A comprehensive summary of the optimized MS/MS parameters for each target analyte, including precursor ions, product ions, and collision energy, is presented in Table S1. Representative chromatograms of the four HNQs compounds are shown in Figure S1.

| **Table S1 MRM mode detection parameters of the target compound** | | | | | |
| --- | --- | --- | --- | --- | --- |
| **Compound** | **Molecular ion** | **Product ion** | **DP（V）** | **CE（V）** | **Retention time（min）** |
| MCNQ | 192.9 | 35 | -56 | -32 | 6.6 |
|  | 194 | 37 | -56 | -32 |  |
| MBNQ | 235.9 | 78.8 | -27 | -18 | 7.5 |
|  | 238 | 81 | -27 | -18 |  |
| DCNQ | 225.9 | 35 | -22 | -51 | 8.8 |
|  | 228 | 37 | -22 | -51 |  |
| DBNQ | 315.7 | 78.9 | -51 | -32 | 9.5 |
|  | 316 | 81 | -51 | -32 |  |


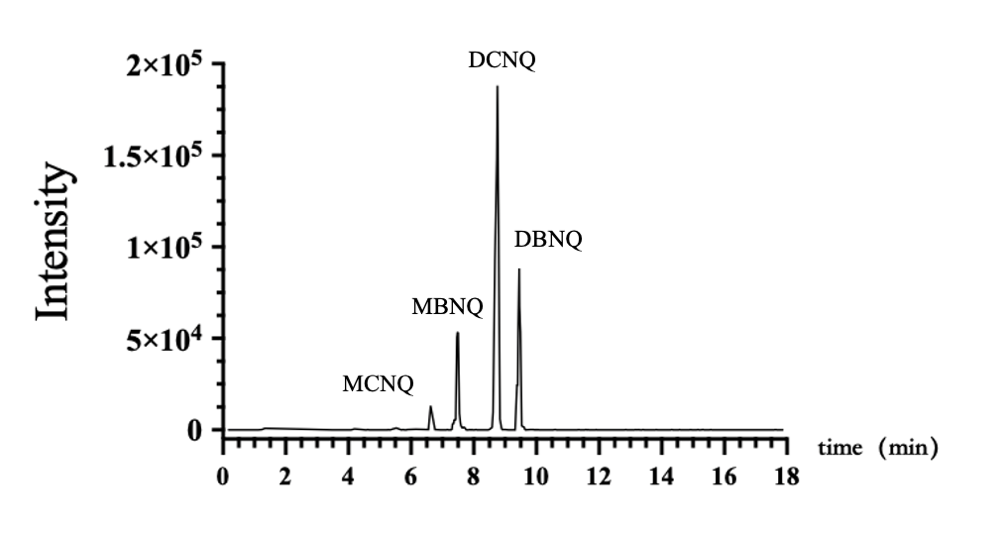


**Figure S1 MRM chromatograms of four HNQs standard solutions**

**TextS4 Quality control**

1. **Working curves, limits of detection, and limits of quantification**

To ensure accurate quantification across the expected concentration ranges in drinking water, separate calibration curves were established for the target compounds based on their observed abundance. For MCNQ and MBNQ, which were present at relatively high concentrations, calibration standard solutions were prepared at six concentration levels: 1, 2, 5, 10, 50, and 100μg/L. For DCNQ and DBNQ, which occurred at lower levels, a more sensitive calibration range was employed, consisting of 0.2, 0.5, 1, 2, 5, and 10μg/L. All calibration standards were prepared in appropriate solvent matrices and subjected to the identical liquid-liquid extraction pretreatment procedure as the actual water samples, followed by HPLC-MS/MS analysis.

As presented in Table S2, all analytes showed excellent linear responses within their respective working concentration ranges, with correlation coefficients (R²) of 0.99 or higher, confirming the suitability of the method for quantitative analysis. The method detection limits (MDL) and method quantification limits (MQL) were determined based on signal-to-noise (S/N) ratios of 3 and 10, respectively, from triplicate measurements of spiked samples, with relative standard deviations (RSD) maintained below 20%. The calculated MDL for the four HNQs compounds ranged from 0.02ng/L to 0.2ng/L, and the corresponding MQLs ranged from 0.07ng/L to 0.67ng/L (Table S2). These low detection and quantification limits indicate that the method possesses high sensitivity and is capable of reliably determining trace levels of HNQs in drinking water samples.

| **Table S2 Working curves, method detection limit, and the method quantification limit** | | | | | |  |
| --- | --- | --- | --- | --- | --- | --- |
| **material** | **Linear range（μg/L）** | **curves** | **R^2^** | **MDL（ng/L）** | **MQL（ng/L）** | |
| MCNQ | 1-100 | y = 3233.5x - 2656.1 | 0.9986 | 0.20 | 0.67 | |
| MBNQ | 1-100 | y = 61942x –32386 | 0.9995 | 0.03 | 0.12 | |
| DCNQ | 0.2-10 | y = 275696x + 5220.5 | 0.9957 | 0.02 | 0.08 | |
| DBNQ | 0.2-10 | y = 462247x – 48620 | 0.9962 | 0.02 | 0.07 | |

1. **Recovery and precision**

The accuracy of the developed method was assessed by performing spike recovery experiments at three concentration levels (low, medium, and high) corresponding to the expected range of HNQs in drinking water. As presented in Table S3, the mean recoveries for the four target compounds ranged from 95.0% to 103.0%, indicating satisfactory accuracy and precision of the extraction and analysis procedure. Method precision was evaluated in terms of both repeatability (intra-day) and reproducibility (inter-day). The intra-day precision RSD was calculated to be 0.2%-1.9%, while the inter-day precision RSD was 1.1%-4.3%. Both values were below the acceptable threshold of 5%, demonstrating excellent analytical stability and reliability of the method for routine application. Detailed precision data are presented in Table S4.

| **Table S3 The recoveries of the LLE-HPLC-MS/MS method for the analysis of the HNQs** | | | | | | | |  |
| --- | --- | --- | --- | --- | --- | --- | --- | --- |
| **Compound** | **Recovery（RSD，n=3,** %**）** | | | | | |  |  |
|  | **0.2μg/L（RSD）** | **1μg/L（RSD）** | | **5μg/L（RSD）** | | **10μg/L（RSD）** | | |
| MCNQ | / | 97.0%（0.11） | 95.5%（0.03） | | 96.3%（0.05） | |  |  |
| MBNQ | / | 88.0%（0.10） | 98.5%（0.02） | | 103.0%（0.05） | |  |  |
| DCNQ | 99.1%（0.06） | 95.0%（0.16） | 96.9%（0.04） | | / | |  |  |
| DBNQ | 95.0%（0.10） | 99.0%（0.14） | 93.1%（0.03） | | / | |  |  |

| **Table S4 Inter-day and intra-day precision of the method** | | | | | | | | | | | | |  |
| --- | --- | --- | --- | --- | --- | --- | --- | --- | --- | --- | --- | --- | --- |
| **Compound** | | **intra-day precision**  **(RSD, %, n=6)** | | | |  | **inter-day precision**  **(RSD, %, n=6)** | | | | | | |
|  |  | **0.2μg/L** | **1μg/L** | **5μg/L** | **10μg/L** |  | **0.2μg/L** | | **1μg/L** | | **5μg/L** | **10μg/L** | |
| MCNQ | / | | 4.3 | 1.1 | 2.2 |  | / | 1.9 | | 1.3 | | 1.1 | |
| MBNQ | / | | 3.3 | 2.8 | 1.8 |  | / | 1.2 | | 1.7 | | 0.6 | |
| DCNQ | 3.5 | | 2.3 | 1.4 | / |  | 1.5 | 0.7 | | 0.3 | | / | |
| DBNQ | 2.3 | | 1.7 | 1.1 | / |  | 0.6 | 0.5 | | 0.2 | | / | |

**TextS5 RNA Extraction and Quantitative Reverse Transcription PCR (qRT-PCR)**

Total RNA was isolated from HepG2 cells using the RNAeasy Animal RNA Extraction Kit (spin column format) purchased from Biyuntian, following the manufacturer's protocol. The concentration and purity of the extracted RNA were determined by measuring absorbance at 260 nm and 280 nm using a NanoDrop 2000 spectrophotometer (Thermo Scientific). Samples with A260/A280 ratios between 1.8 and 2.1 were considered acceptable for subsequent analysis. For each sample, 1 μg of total RNA was reverse transcribed into complementary DNA (cDNA) using the HiScript III RT SuperMix for qPCR (+gDNA wiper) kit (Vazyme), which includes a genomic DNA removal step. Quantitative real-time PCR (qPCR) was performed on the resulting cDNA using the SupRealQ Purple Universal SYBR qPCR Master Mix (U+) kit (Vazyme). Gene-specific primer pairs were designed based on sequences obtained from the GenBank database. Primer specificity was verified using the NCBI Primer-BLAST tool to ensure no cross-reactivity with non-target sequences. The sequences of all primers used in this study are provided in Table S5. The β-actin was used as an internal reference to normalize gene expression levels. Relative quantification of target gene expression was performed using the comparative threshold cycle (Ct) method. The fold change in gene expression between treatment and control groups was calculated using the 2^−ΔΔCt^ formula, with the control group serving as the calibrator.

| **Table S5. Primer sequences for RT-qPCR of HNQs treated HepG2 cells** | | | |
| --- | --- | --- | --- |
| **Gene name** | | **Forward (5’-3’)** | **Reverse (5’-3’)** |
| *IPMK* | AGGACAACTGTCAGACACAGA | | TGACGCGCATACATCTTGGAC |
| *AMPK* | AACAAGTTGTGGCTCACCCA | | AGAATCAGGTGGGCTTGTCG |
| *SIRT1* | TAGCCTTGTCAGATAAGGAAGGA | | ACAGCTTCACAGTCAACTTTGT |
| *PGC-1α* | AAGCACTTCGGTCATCCCAG | | GTAGCTGTCATACCTGGGCC |
| *NRF1* | TCAGAATTGCCAACCACGGT | | CCAATGTCACCACCTCCACA |
| *TFAM* | GGTGGTTTTCATCTGTCTTGGC | | CGTTGTTTCTTTATTGTGCGACG |
| *P62* | GGAGCACGGAGGGAAAAGAA | | GCCGCTCCGATGTCATAGTT |
| *Beclin-1* | CGTCCAACAACAGCACCATG | | TTTCCACGTCTTCCAGCTCC |
| *ATG5* | CGAGATGTGTGGTTTGGACG | | CCACAGGACGAAACAGCTTC |
| *β-actin* | TGTCCACCTTCCAGCAGATGT | | AGCTCAGTAACAGTCCGCCTAGA |

**Table S6 Exposure concentrations of HNQs in experimental groups**

| **Table S6 Exposure concentrations of HNQs in experimental groups (ng/L)** | | | | | | |
| --- | --- | --- | --- | --- | --- | --- |
| **Compound** | **1**$\boldsymbol{\times}$ | **10**$\boldsymbol{\times}$ | **50**$\boldsymbol{\times}$ | **100**$\boldsymbol{\times}$ | **500**$\boldsymbol{\times}$ | **1000**$\boldsymbol{\times}$ |
| MCNQ | 13.05 | 130.5 | 652.5 | 1305 | 6525 | 13050 |
| DCNQ | 2.05 | 20.5 | 102.5 | 205 | 1025 | 2050 |
| MBNQ | 13.89 | 138.9 | 694.5 | 1389 | 6945 | 13890 |
| DBNQ | 1.38 | 13.8 | 69.0 | 138 | 690 | 1380 |

**Figure S2 Water plant treatment process**

**
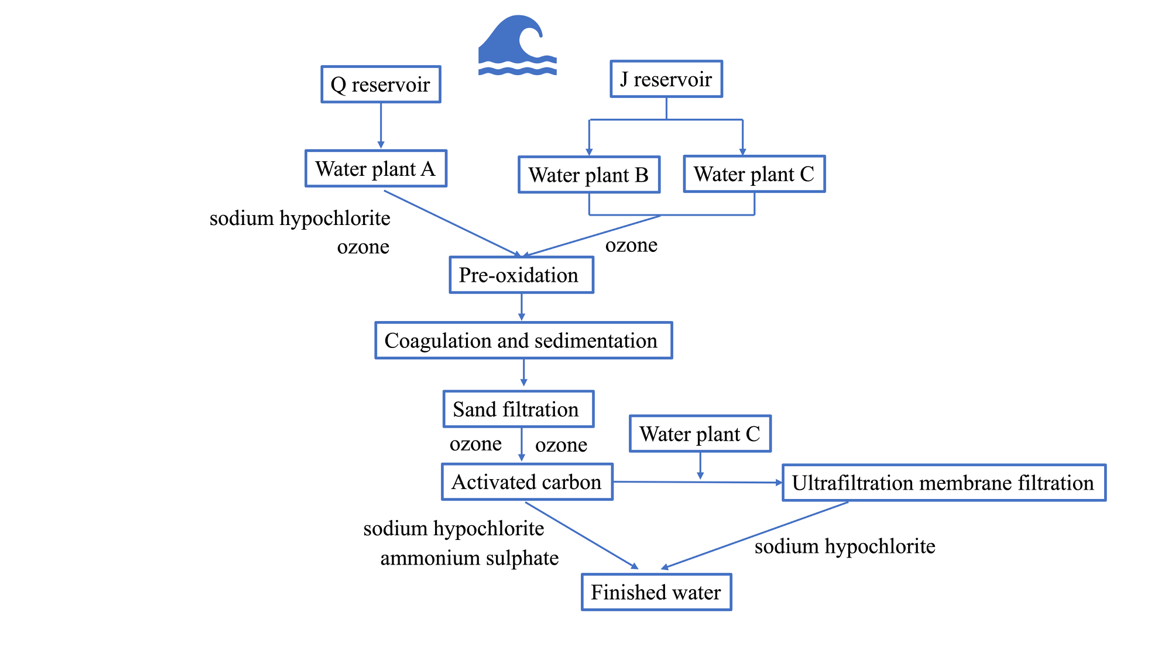
**

**Figure S2 Drinking water treatment process flow diagram of three DWTPs**

**Figure S3 Water sample preparation optimization**


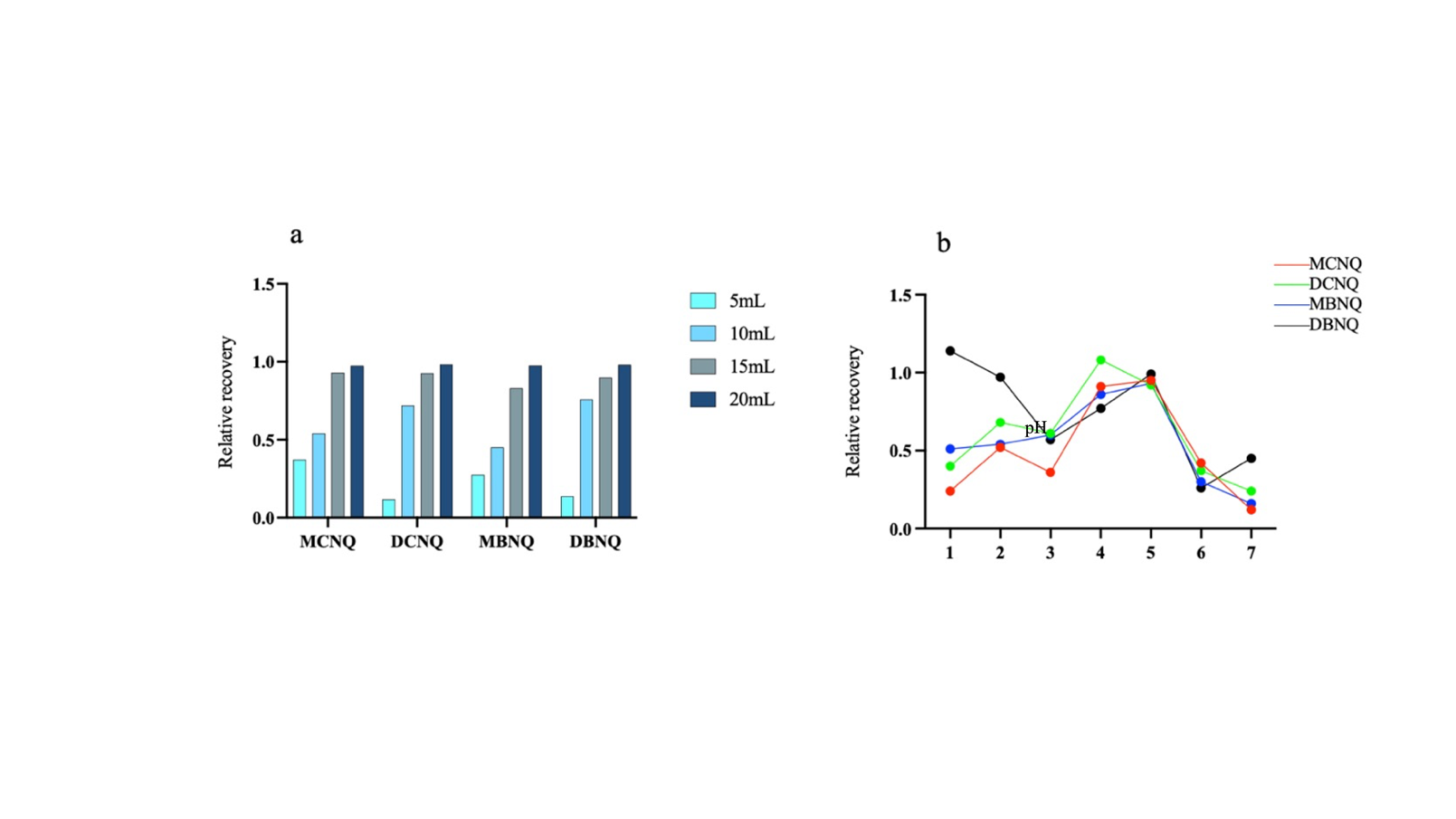


**Figure S3 The recoveries of four HNQs using different volumes of n-hexane extraction and different pH conditions**

**a: Recovery of using different volumes of n-hexane extraction; b: Recovery at different pH conditions**
